# High-fat diet, but not duration of lactation, increases mammary gland lymphatic vessel function and subsequent growth of inflammatory breast cancer cells

**DOI:** 10.1007/s10911-023-09548-8

**Published:** 2023-10-06

**Authors:** Wintana Balema, Janelle Morton, Richard A. Larson, Li Li, Fred Christian Velasquez, Natalie W. Fowlkes, Savitri Krishnamurthy, Bisrat G. Debeb, Eva Sevick-Muraca, Wendy A. Woodward

**Affiliations:** 1 MD Anderson UTHealth Houston Graduate School of Biomedical Sciences, Houston, TX USA; 2 Department of Experimental Radiation Oncology, The University of Texas MD Anderson Cancer Center, Houston, TX USA; 3 The Morgan Welch IBC Clinic and Research Program, The University of Texas MD Anderson Cancer Center, Houston, TX USA; 4 The University of Texas Health Science Center, Institute of Molecular Imaging, Center for Molecular Imaging, Houston, TX USA; 5 Department of Veterinary Medicine and Surgery, UT MD Anderson Cancer Center, Houston, TX USA; 6 Department of Pathology, UT MD Anderson Cancer Center, Houston, TX USA; 7 Department of Breast Medical Oncology, The University of Texas MD Anderson Cancer Center, Houston, TX USA; 8 Department of Breast Radiation Oncology, UT MD Anderson Cancer Center, Houston, TX USA

**Keywords:** High fat diet, IBC, Inflammatory breast cancer, Lymphatic pulsing, Podoplanin, Mammary gland, Weaning, Lactation, Pregnancy, Microenvironment, IBA1, CD163, PoEM

## Abstract

Inflammatory breast cancer (IBC) presents as rapid-onset swelling and breast skin changes caused by tumor emboli in the breast and breast skin lymphatics. IBC has been linked with obesity and duration of breastfeeding, but how these factors affect IBC tumor progression is not clear. We modeled the simultaneous effects of diet and weaning in mice on in vivo lymphatic function; on IBC tumor growth; and on aspects of the mammary gland microenvironment before and after IBC (SUM149) xenograft inoculation. We hypothesized that weaning status and diet would have synergistic effects on lymphatic function and the breast microenvironment to enhance IBC tumor growth. Changes in lymphatic structure and function were characterized with in vivo near-infrared fluorescence (NIRF) imaging. Mice were fed either a high-fat diet (HFD; 60 kcal%) or a normal/low-fat diet (LFD; 10 kcal%), bred twice, and subjected to either normal-duration nursing (NW) or forced weaning (FW). SUM149 IBC tumors were implanted at 14 months; images were obtained before and after implantation. Multiparous mice fed HFD showed increased pre-tumor lymphatic pulsing in both the FW and NW groups relative to mice fed LFD. HFD promoted tumor growth independent of weaning time (*P* = 0.04). Pre-tumor lymphatic pulsing was associated with tumor volume at 8 weeks (*P* = 0.02) and was significantly correlated with expression of the lymphatic tracking ligand CCL21 (*P* = 0.05, Table 1). HFD significantly increased the numbers of monocyte-derived IBA1^+^, CD163^+^, and CD11c^+^ cells (*P* < 0.0001, *P* < 0.0001, *P* = 0.0005) in the contralateral, non-tumor-bearing mammary gland. Numbers of lymphangiogenic podoplanin^+^/IBA1^+^ macrophages were increased in the ducts of HFD and FW mice (all *P* < 0.003). HFD in nulliparous mice had a similar increase in lymphatic pulsing at 14 weeks (*P* = 0.006), indicating that this functional change was independent of parity. We conclude that HFD induced increases in mammary gland lymphatic function, assessed as pulsing rate before tumor initiation, and correlated with inflammation in the mammary gland and increased SUM149 tumor growth. The relationship between diet, lymphatic pulsing, and tumor growth warrants further investigation.

**Supplementary Information:**

The online version contains supplementary material available at 10.1007/s10911-023-09548-8.

## Introduction

Inflammatory breast cancer (IBC) is an aggressive form of breast cancer that presents as rapid breast swelling and skin color changes thought to be due to congested lymphatics in the breast and breast skin [1, 2]. These symptoms are partly attributed to IBC tumor emboli clogging dermal lymphatics around the breast, causing lymphovascular skin invasion (LVSI) [3, 4]. In many IBC cases, metastatic spread has an initial lymphatic-based pattern including mediastinal or contralateral lymph nodes, suggesting that lymphatic vessels can attract and facilitate the spread of the tumor [5–7]. This pattern highlights the importance of understanding lymphatic development and function in mediating the poor outcomes associated with this disease. Emerging evidence suggests that the breast microenvironment can induce and promote IBC symptoms including LVSI and its distinctive, diffuse growth pattern [8, 9]. We previously reported that in an in vivo SUM149 model, mesenchymal stromal cells signal via macrophages to promote skin invasion by IBC (SUM149) cells [8]. Individual risk factors for breast cancer are well known to affect the mammary gland microenvironment [10, 11]; however, the factors in the breast microenvironment that contribute to LVSI and especially lymphatic function have not been well studied.

Atkinson et al. reported in a single-institution case–control study that obesity and lack of breast feeding were associated with aggressive subtypes of IBC [10]. Genomic analyses of breast tissues adjacent to demonstrated enrichment for gene signatures associated with involution after weaning, suggesting that involution biology can persist in the breast tissues for years and could contribute to the development of this aggressive breast cancer [11]. We sought here to investigate the potential synergy between IBC risk factors, focusing on obesity and “weaning time” (i.e., duration of nursing) to create a pro-IBC, lymphatic-rich mammary stroma before tumor initiation. Recognizing the critical role of lymphatic function in IBC and the lack of data specifically examining function, we used near-infrared fluorescence (NIRF) imaging of mammary-draining fluorescent dye to study the synergy of risk factors on lymphatic function.

We report that a high-fat diet (HFD) was associated with increased mammary lymphatic pulsing and IBC (SUM149) tumor growth in mice. The HFD significantly increased inflammation of specific mammary duct-infiltrating macrophages and other cells, including a lymphangiogenic subset of macrophages, podoplanin^+^ (PDPN^+^) macrophages (PoEMs), in lymphatic vessels and mammary ducts of HFD and force-weaned mice. These studies elucidate the role of diet in lymphatic function and IBC (SUM149) progression in this model and provide new hypothesis-generating findings regarding the synergy between weaning and diet in IBC for further study.

## Methods

### Mice

All animal experiments were conducted in accordance with institutional animal regulations and American Association for Laboratory Animal Science guidelines. Balb/c SCID/Beige mice were purchased from The Jackson Laboratory (Bar Harbor, ME) and maintained in a pathogen-free mouse facility.

#### Experiment 1 (nulliparous animals)

Ten female mice were started on either a HFD (60 kcal%, *n* = 5) (catalogue no. D12492i, Research Diets, Inc) or a low-fat diet (catalogue no. D12450Bi, LFD) (10 kcal%, *n* = 5) when they were 3 weeks old and maintained on this diet through imaging at 14 weeks (Supplementary Fig. 1). One animal in each group died without obvious pathology prior to imaging.

#### Experiment 2 (multiparous animals)

At 3 weeks of age, 20 mice were initiated on either a HFD (60 kcal%, *n* = 10) (Research Diets, Inc) or LFD (10 kcal%, *n* = 10). These mice were then bred and impregnated twice in succession, and each diet group was further randomized by weaning time as either nurse- or naturally-weaned (NW, full 21 day weaning cycle), versus force-weaned (FW, pups removed at day 1 after each pregnancy) (10 mice each; 5 mice in each diet + weaning group). After the second imaging timepoint at 14 months, the mice were inoculated with IBC (SUM149) tumors, the tumor volume was monitored and measured weekly using a caliper. Tumor volume was calculated; 0.5(length x width x width) (Supplementary Fig. 2).

### In vivo near-infrared fluorescence lymphatic imaging

Mice in the treatment groups described above were transferred from MD Anderson Cancer Center to The University of Texas Health Science Center for imaging and were maintained there until euthanasia. At each imaging session, depilatory cream (Nair; Church & Dwight Co., Inc) was used to remove hair from the skin over the #4 and #9 mammary glands. Mice were then anesthetized with isofluorane, placed on a warming pad (37 °C), and NIRF images were obtained and quantified as follows. A 10-µL volume of indocyanine green (ICG) dye (Akron, Inc.) was subdermally injected near the areola into the ventral #4 and #9 mammary fat pad. Fluorescence images of the ventral vessels and proximal left and right vessels were acquired immediately and then continuously over the ensuing 8 min by using an electron-multiplying charge-coupled device (EMCCD) camera (PhotonMax 512B, Princeton Instruments, Tucson, AZ), with image acquisition by V +  + software (Digital Optics, Aukland, New Zealand). Matlab (The MathWorks Inc., Natick, MA) and ImageJ (National Institutes of Health, Washington, DC) were used to reveal lymphatic contractility. Two fixed regions of interest (ROIs) in fluorescent lymph channels were defined on fluorescence images for right and left vessels, one each for each ventral vessel. The mean of the fluorescence intensity within each ROI in each fluorescence image was then calculated and plotted as a function of imaging time to provide counts per minute [12]. Animals were sacrificed and tumor and mammary gland tissues collected.

### Multiplex immunofluorescence (IF) staining

Formalin-fixed, paraffin-embedded (FFPE) blocks of tumor and contralateral normal mammary gland collected from the mice after the post-tumor imaging session were submitted for tissue microarray construction, multiplex panel optimization, and testing. A pathologist (NF) reviewed hematoxylin and eosin (H&E) stains of each FFPE section to identify regions of interest in the mammary gland that would capture tissue heterogeneity. Subsequently, a cylindrical core punch biopsy was obtained from both a vimentin-high and vimentin-low region of each FFPE block (two total) to represent two biological replicates from this bipotent (maintains luminal and mesenchymal marker expressing epithelial cells in culture) xenograft model, where expression of vimentin would identify more mesenchymal tumor regions and vimentin-low regions would represent epithelial outgrowths. Cores were then transferred to the recipient block, which was further sectioned by using a microtome into 4-µm-thick sections. Each mammary gland section had two replicates from intentionally distinct tissue regions. All sections were subjected to chromogenic immunohistochemical staining to validate and optimize targets by using a Leica Bond RX autostainer. Antibody target stains were grouped in two panels: Panel A targets were CD31 (ABCAM, cat #28,364), IBA1 (ABCAM, cat #178,847), alpha-smooth muscle actin (αSMA) (ABCAM, cat #5694), podoplanin (PDPN; Invitrogen, cat #29,742), vimentin (Cell Signaling, cat #5741), and KRT19 (ABCAM, cat #52,625). Panel B targets were CD163 (ABCAM, cat #182,422), CD11b (ABCAM, cat #133,357), CCR7 (Invitrogen, cat #MA5-31,992), CD11c (Cell Signaling, cat #97,585), CCL21 (Invitrogen, cat #114,959), and KRT19 (ABCAM, cat #52,625). Immunohistochemically stained samples were scanned with an Aperio AT2 (Leica Biosystems, Wetzler, Germany). Multiplex immunofluorescence (IF) staining was done with an Opal 7-Color Kit for multiplex immunohistochemical analysis (Akoya Biosciences, Marlborough, MA) on a Leica Bond Rx autostainer. The stained slides were subsequently scanned with a Leica VERSA 8 (Leica Biosystems, Wetzler, Germany), and images captured with Leica ImageScope software.

### Quantification and analysis of multiplex IF-stained specimens

Tissue microarray slides with cores containing mouse normal mammary gland and tumor sections were stained for Panel A and Panel B markers for multiplex IF and scanned with a Leica VERSA 8 whole-slide fluorescent digital scanner as described above. IF was quantified with the image analysis tool in ImageScope v 12.4.3, and a cellular IF algorithm was selected and modified for each panel. Four different algorithms were tuned for each panel and tissue type. First, the cellular IF algorithm was tuned to segment cellular nuclei to ensure accurate identification and quantification of 4′,6-diamidino-2-phenylindole (DAPI) -stained nuclei. Next, the algorithm was tuned for positive fluorescence intensity to quantify the tissue expression of each marker while minimizing non-specific background staining or autofluorescence. Once the tuning was completed for the individual markers, co-expression classes of selected markers within cells were created and included in the algorithm quantification. To evaluate the lymphatic vessel and mammary duct cellular environment, each vessel and duct were individually manually annotated in the images of the mammary gland cores for structural and functional analysis with these algorithms. Ductal expression was quantified as positive cells per annotated duct structure and expression per duct for each duct was plotted from all mice as a group.

### Statistical analyses

Heterogeneity in tumors observed from H&E and vimentin staining led to our choosing to use both replicates from each tumor specimen, without averaging, for statistical analysis. GraphPad Prism was used to plot graphs and perform *t* tests and one-way analyses of variance, assuming independent samples. *P* values of < 0.05 were considered to indicate significant differences. Pearson’s correlations were performed with SPSS (version 23).

## Results

### High-fat diet significantly increased lymphatic pulsing in nulliparous and multiparous mice independent of weaning status

**Fig. 1:**
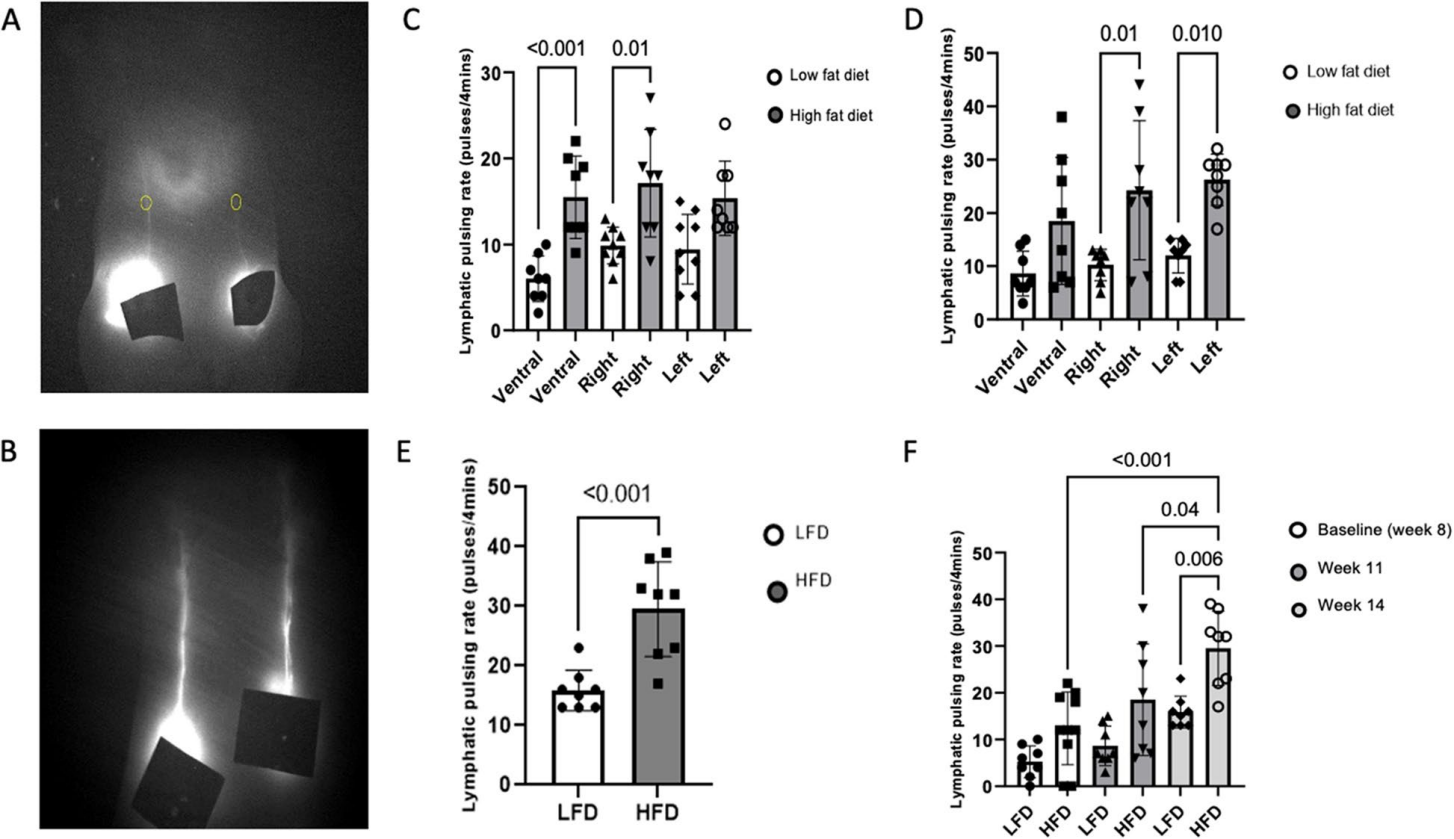
High-fat diet increases mammary lymphatic pulsing relative to a low-fat diet in nulliparous mice. NIRF imaging. All mice had two ventral, two left and two right measurements, *N* = 5 per group, however one mouse in each group died prior to imaging. **A**, **B** Representative in vivo near-infrared fluorescence (NIRF) images of ventral dermal lymphatic vessels above mammary gland #4 and #9 after subdermal injection of indocyanin green in mice fed a low-fat diet (LFD, A) or a high-fat diet (HFD, B). **C** Lymphatic contractile rate by diet at week 8, (**D**) week 11, (**E**) week 14 only ventral pulsing detected. **F** Lymphatic contractile activity over time

To investigate the effect of HFD on lymphatic function in the mammary gland, we measured lymphatic pulsing using NIRF in nulliparous mice started on HFD or LFD at 3 weeks of age. The average weight of the mice at the time of the first imaging in the LFD group was 21.3 g and that in the HFD group was 26.8 g (*P* = 0.005). Representative ventral NIRF images in the LFD and HFD groups are shown in Fig. 1A [for the LFD group] and Fig. 1B [for the HFD group]. At week 8, the lymphatic pulsing activity was significantly increased in the ventral and right dermal lymphatic vessels in the HFD mice Fig. 1C *P* < 0.001 ventral, HFD vs LFD; and *P* = 0.01 right, HFD vs LFD). At week 11, the lymphatic pulsing activity was significantly increased in the right and left dermal lymphatic vessels in the HFD mice (Fig. 1D, *P* = 0.01 for both). At week 14, no lymphatic pulsing was observed on the left or right; however, ventral lymphatic pulsing was increased significantly in the HFD mice at that time (Fig. 1E, *P* < 0.001 vs LFD). The lymphatic pulsing activity increased significantly in the HFD group at each time point. Average ventral lymphatic contractile frequency for LFD and HFD at 8, 11, and 14 weeks were (LFD) 1.25, 2.16, and 3.97 pumps/min vs (HFD) 2.94, 4.63, and 7.06 pumps/min (Fig. 1F).

**Fig. 2:**
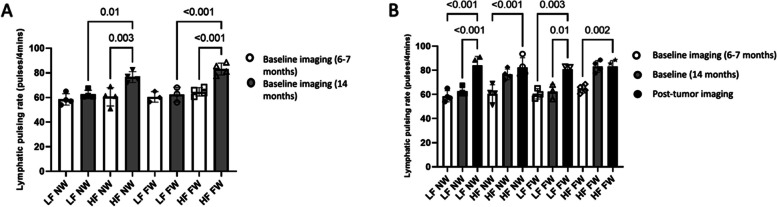
HFD increases lymphatic pulsing from mammary gland–draining lymphatics in multiparous mice to a degree similar to that induced by tumor initiation. **A** Lymphatic pulsing by diet and weaning status (*N* = 5 animals per group, fewer than five points reflects animal loss prior to imaging), at 6–7 months and 14 months. (**B**) Lymphatic pulsing pre and post tumor inoculation and growth

Next, to investigate the synergistic effects of HFD and lactation/weaning time on dermal lymphatic pulsing activity, multiparous mice that had been fed either the HFD or LFD were abruptly (forced) weaned or naturally (nursed) weaned (Fig. 2). Imaging included only the ventral lymphatics in these mice. The initial NIRF imaging occurred at 6 months of age but was interrupted/incomplete due to the COVID-19 pandemic; it was completed after the shut-down, compromising the data point, and thus was repeated a single imaging session baseline at 14 months. Mice were maintained on the diets without interruption during this time. In multiparous mice, lymphatic pulsing before tumor inoculation in HFD force-weaned (HF FW) and HFD nurse-weaned (HF NW) animals was increased compared to LF FW and LF NW (Fig. 2A, *P* < 0.001 and *P* = 0.01); whether mice were allowed to wean did not affect lymphatic pulsing activity in these mice (Fig. 2A, *P* = not significant [NS]).

### High-fat diet significantly increased IBC (SUM149) tumor growth in multiparous mice

**Fig. 3:**
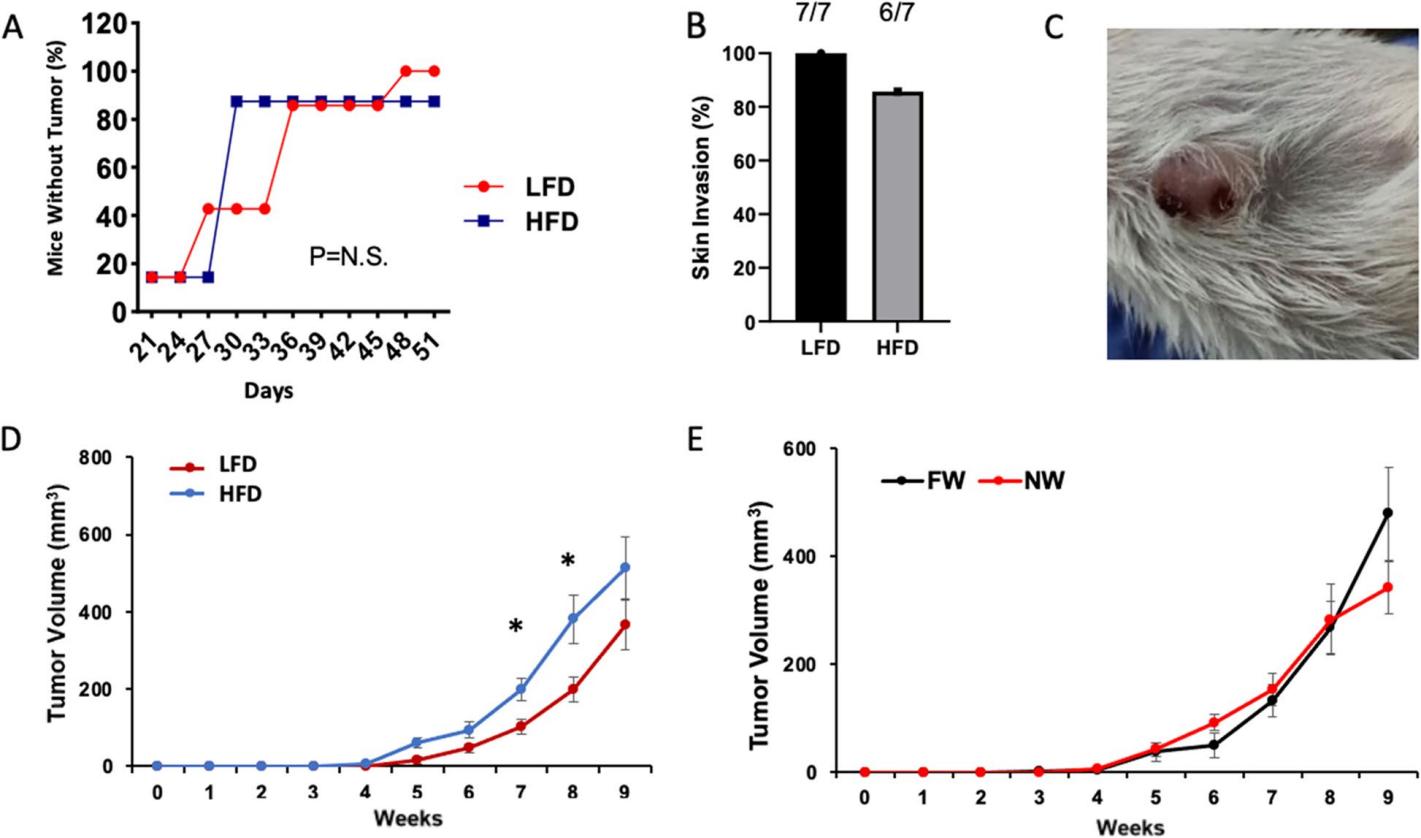
High-fat diet promotes IBC (SUM149) tumor growth in SCID/beige multiparous mice. **A** Percent of mice with tumor over time by diet group (LFD *N* = 7, HFD *N* = 7). **B** Skin symptoms by diet group. **C** Photograph of representative skin symptoms. **D**, **E** Tumor growth delay by diet (*P = 0.04 at 7 weeks, 0.05 at 8 weeks)

To determine how risk factor–primed microenvironments influenced tumor growth, lymphatic activity, and vasculature after the orthotopic inoculation of SUM149 tumor cells into the #4 mammary gland fat pad of these mice, post-tumor-inoculation NIRF lymphatic pulsing images were obtained at 16 months (~ 8 weeks after tumor initiation). At 16 months, the presence of tumor was associated with significantly increased dermal lymphatic pulsing activity compared with the baseline (pretumor) pulsing activity in LFD groups, independent of weaning status (Fig. 2B), whereas HFD groups had already achieved virtually the same degree of increase before tumor initiation. Neither diet (Fig. 3A) nor weaning status (not shown) affected the percent of mice with tumors. In addition, IBC-like skin symptoms, scored as hair loss with bleeding or skin blisters and evident tumor growth into the skin, was present in 13 of the 14 mice in which tumors developed, and thus was not significantly different across the groups (Fig. 3B, C). Notably, this skin symptom incidence in these multiparous mice was unexpectedly higher than was previously reported for nulliparous mice in this model (25%) [9] and may indicate that pregnancy affects skin symptoms more than duration ofnursing, although the role of age or other factors was not evaluated and skin symptoms in mice, while comparable to findings clinically used for patient diagnosis may not be a comparable endpoint in mice. HFD-fed mice had modestly enhanced tumor growth relative to LFD-fed mice (Fig. 3D). However, weaning status did not affect IBC (SUM149) tumor growth (Fig. 3E, *P* = NS). Lymphatic pulsing trended towards correlation with tumor size at 42 days (*P* = 0.08) and was significantly correlated to tumor size at 48 days (*P* = 0.02).

**Fig. 4:**
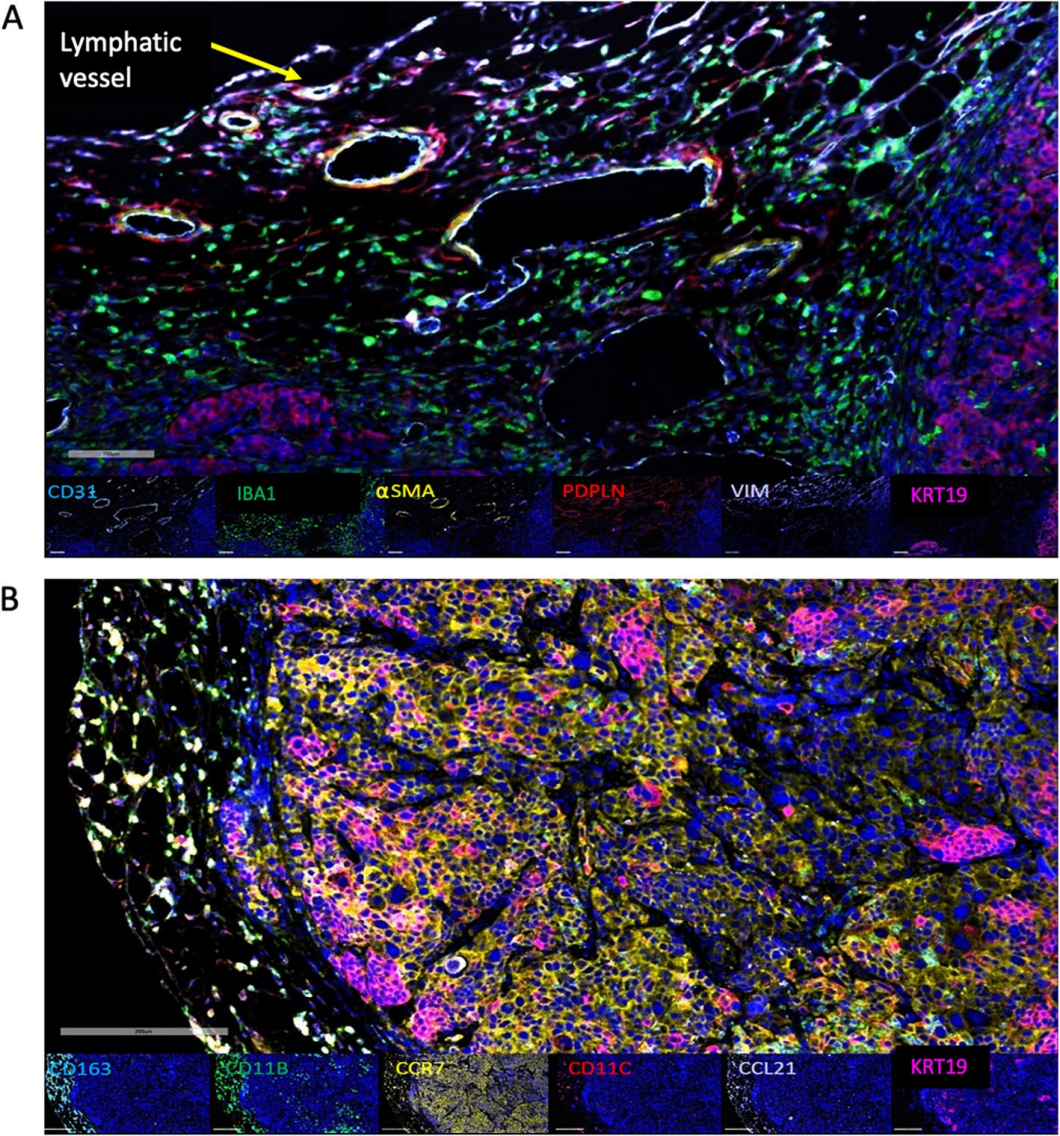
Tissue microarray multiplex immunofluorescence stains of markers from two panels in mammary gland tissue and tumors. **A** Tumor–stroma interface (20X) shows panel A staining: CD31 (teal), IBA1 (green), αSMA (yellow), podoplanin (red), vimentin (white), KRT19 (magenta). Scale bar is 100 μm. **B** Tumor Sect. (20X) shows panel B staining: CD163 (teal), CD11b (green), CCR7 (yellow), CD11c (red), CCL21 (white), and KRT19 (magenta). Scale bar is 200 μm

Considering molecular mediators of lymphatic trafficking, we examined the expression of C–C chemokine ligand 21 (CCL21) and C–C chemokine receptor 7 (CCR7). CCL21 is a ligand for the leukocyte receptor CCR7, which mediates leukocyte homing and trafficking towards lymphatics. We found that tumor size was significantly correlated with the number of cells expressing CCL21 (Representative image Fig. 4A, Correlation, *P* = 0.05, Table 1). Thus, we stained tumors from these mice for CCR7 (Fig. 4B), which might be expected to mediate lymphatic homing to this ligand; we found that CCR7 was expressed in SUM149 tumors and was numerically–but not significantly–increased in the HF FW mice. Of the other markers examined (Supplemental Fig. 3A-E), including podoplanin, only CD31 was significantly increased in tumors from HFD mice. There were no significant differences between NW and FW mice (Supplemental Fig. 3 F-J). Interestingly, CCR7 staining in tumors was significantly correlated with pre-tumor lymphatic pulsing in FW mice but not in NW mice (Supplementary Fig. 3 K-N).

**Table 1:** Correlation between lymphatic pulsing, CCL21/CCR7 IF positive cells, and tumor growth and lymphangiogenesis

		CCL21	Lymphatic Pulsing
Tumor Size (Week 8)	Pearson Correlation	0.16	0.648
	Significant (2-tailed)	0.61	**0.02**
	N	13	13
CCL21	Pearson Correlation	1	0.557
	Significant (2-tailed)		**0.05**
	N	13	13
Lymphatic Pulsing	Pearson Correlation	0.557	1
	Significant (2-tailed)	**0.05**	
	N	13	13
CCR7	Pearson Correlation	0.708	0.4
	Significant (2-tailed)	**0.007**	0.1
	N	13	13

### High-fat diet–induced increase in mammary lymphatic function was independent of lymphatic vessel number

HFD significantly increased lymphatic pulsing activity, independent of weaning status, to a similar extent that tumor initiation did. To determine if this increase in lymphatic functionality was due to increased lymphatic vessel density, we used multiplex IF staining for lymphatic markers and examined the contralateral mammary gland sections from the multiparous mice to determine the number of lymphatic vessels. Multiplex IF staining of the tissue microarrays was successfully completed for the markers in 2 panels, CD31 (endothelial cells), IBA1 (macrophages), αSMA (myofibroblasts), PDPN [a marker of lymphatic endothelial cells], vimentin (marker of the epithelial-to-mesenchymal transition), KRT19 (tumor stem cells), CD163 (anti-inflammatory macrophages), CD11b (macrophages, NK cells), CCR7 (dendritic cells, NK cells, T cells), CD11c (dendritic cells), and CCL21 (T cells),in the tumor and mammary gland tissues (Fig. 4, Supplementary Fig. 3). Lymphatics were identified and annotated manually based on PDPN staining using the entire stained core, and labeled CCL21 cell counts were exported by using ImageScope algorithms (Table 1).

**Fig. 5:**
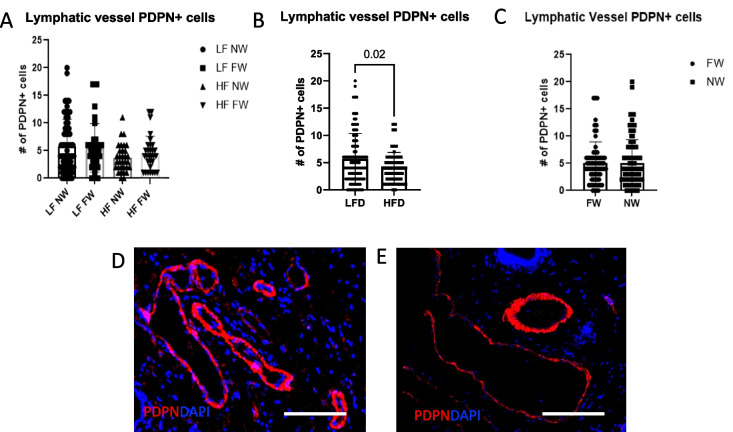

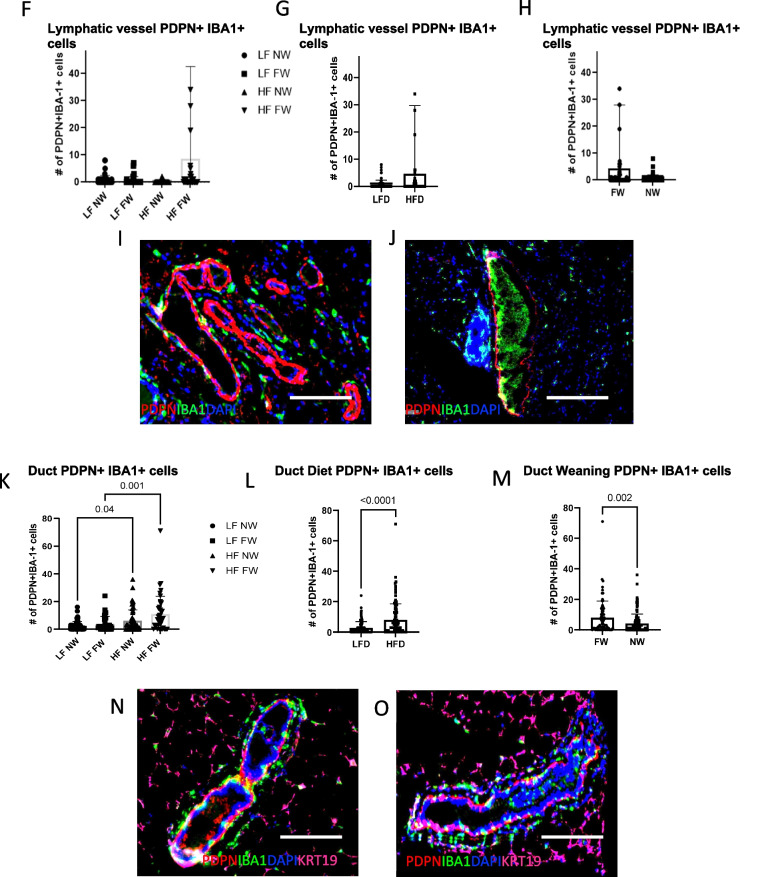
High-fat diet enhances lymphatic function independent of lymphatic vessel numbers. Lymphatic vessels were annotated manually based on podoplanin [PDPN]-expression. Ducts were annotated based on KRT19 expression. Each point represents the number of positive cells within an annotated structure (thus the total number of points represents the number of lymphatic or ductal structures summed over all mice in the group). **A** Total number of PDPN^+^ cells localized to annotated lymphatics across all mice within each group (LF, low fat, HF high fat, NW, nurse weaned, FW, force weaned). **B** Total number of PDPN^+^ cells within annotated lymphatics across mice in HFD versus LFD groups. **C** Total number of PDPN^+^ cells within annotated lymphatics by weaning status. **D**, **E**. Multiplex IF images show increased density of PDPN^+^ cells in lymphatic vessels (red) in mammary gland sections from LFD mice (**D**) Relative to the HFD group (**E**). **F**, **G**, **H** Co-expression of PDPN and IBA1^+^ per annotated lymphatic. **I**, **J** IF images show infiltration of PDPN^+^IBA1^+^ macrophages in the mammary-gland lymphatic vessels in the HF FW and LF FW groups. **K**, **L**, **M** PDPN^+^IBA^+^ macrophages per annotated mammary ducts based on KRT19 staining. **N** IF images of PDPN^+^IBA1^+^ macrophages in ducts from FW mice (yellow; represents combination of red PDPN and green IBA1) and NW mice. All scale bars are 100 μm

Increased lymphatic function was not associated with an increased number of vessels. No significant differences were found in expression of PDPN across all four treatment groups; in aggregate, the mice fed HFD actually had fewer PDPN^+^ lymphatic cells than the mice fed LFD (Fig. 5A, *P *= N.S.; Fig. 5B, *P *= 0.02). To confirm that the total number of lymphatics was not increased in the mice with increased function, we also assessed lymphatic morphology in the H&E images. The average number of lymphatic vessels identified by manual annotation of H&E-stained sections were not different between groups (Table 2, *P*=NS). Receipt of HFD also led to having decreased PDPN^+^ cell counts in annotated lymphatic vessels compared with receipt of LFD (Fig. 5B, *P*=0.02; representative images shown in Fig. 5D, E).

**Table 2:** Average number of lymphatic vessels in each mammary gland core for each treatment group

Treatment groups	Avg. #Lymphatic vessels	Avg. #Ducts
LF NW	18.3	15.7
LF FW	3.3	10
HF NW	7.7	31.7
HF FW	8	18.7
	*P* = 0.46	*P* = 0.38

### Expression of lymphangiogenic, lympho-invasive PDPN^+^ ductal macrophages (PoEMs) and other mammary duct–associated monocyte-derived cells

**Fig. 6:**
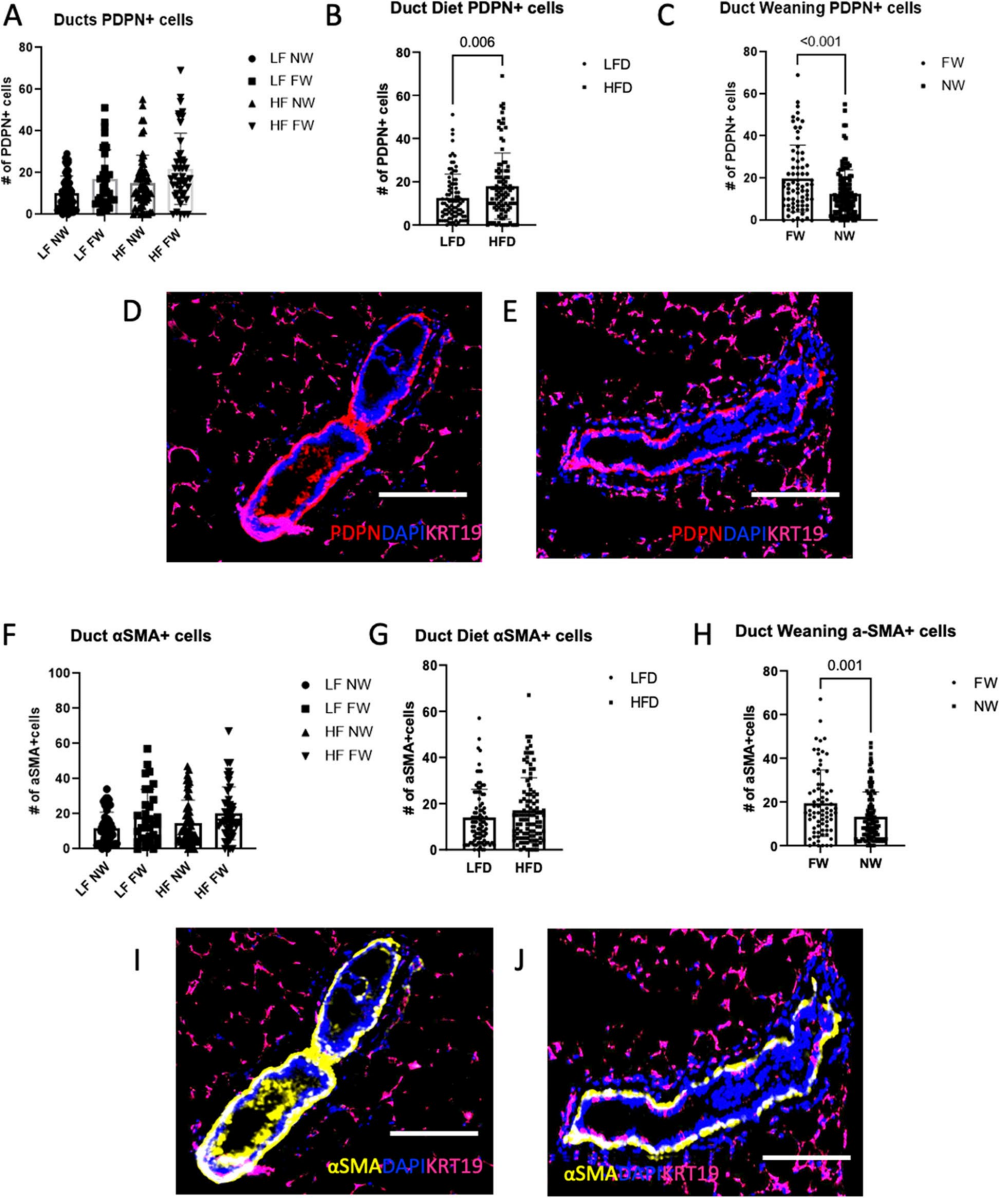
High-fat diet and forced weaning synergistically increase PDPN^+^ ductal cells. **A**, **B**, **C** PDPN^+^ ductal cells. **D**, **E** Immunofluorescence (IF) images show greater numbers of PDPN^+^ (red) cells in the mammary ducts from the FW mice than in the NW mice. **F**, **G**, **H** Numbers of ductal cells expressing alpha-smooth muscle actin (αSMA) per duct (**G**, **H**). **I**, **J** IF αSMA^+^ ductal cells (yellow). All scale bars are 100 μm

Analysis of multiparous tumor-bearing mice also showed that HFD increased markers of inflammation and lymphangiogenesis in the contralateral gland independent of lactation/weaning status. Podoplanin-expressing macrophages, PoEMS, have been recently described as lymphangiogenic and LVSI-promoting (42). Thus, we sought to identify mammary-duct–infiltrating PDPN^+^IBA1^+^ populations within manually annotated epithelial ductal and lymphatic structures. Within lymphatic vessels, the HF FW mice had the highest numeric concentration of PDPN^+^IBA1^+^ cells (Fig. 5F, G, H, I, J, *P* = NS), but the numbers were low overall and not statistically significant. Next, we examined ductal PoEMs. As was the case for the lymphatic vessels, HFD plus forced weaning increased the numbers of ductal PoEMs (Fig. 5K; L, *P* < 0.0001 and Fig. 5M, *P* = 0.002). This increased presence of ductal PDPN+ macrophages in the FW group (relative to the NW group) is illustrated by yellow fluorophores in Fig. 5N and O. Ductal epithelial cells also expressed significantly higher PDPN+ cells in HFD mice (Fig. 6A, NS, 6B, *P* = 0.006), and the FW mice expressed higher ductal PDPN+ cells than the NW mice (Fig. 6C, *P* < 0.001). Figures 6D and E show increased numbers of PDPN+ ductal cells within the mammary ducts in the FW mice versus the NW mice. To determine if the numbers or nature of ducts were different between groups, we evaluated lobular subtypes in these mice (type 1 is 11–15 acini per lobule, type 2 is > 15–50, and type 3 is > 50; type 4 is defined as a terminally differentiated milk-secreting lobule) but found no differences across groups (not shown). Ductal cells expressing αSMA also did not vary across all the treatment groups (Fig. 6F) nor by diet (Fig. 6G, *P* = NS). However, forced weaning led to significant increases in numbers of αSMA+ ductal cells (Fig. 6H, *P* = 0.001). Representative images are shown in Fig. 6I and J.

**Fig. 7:**
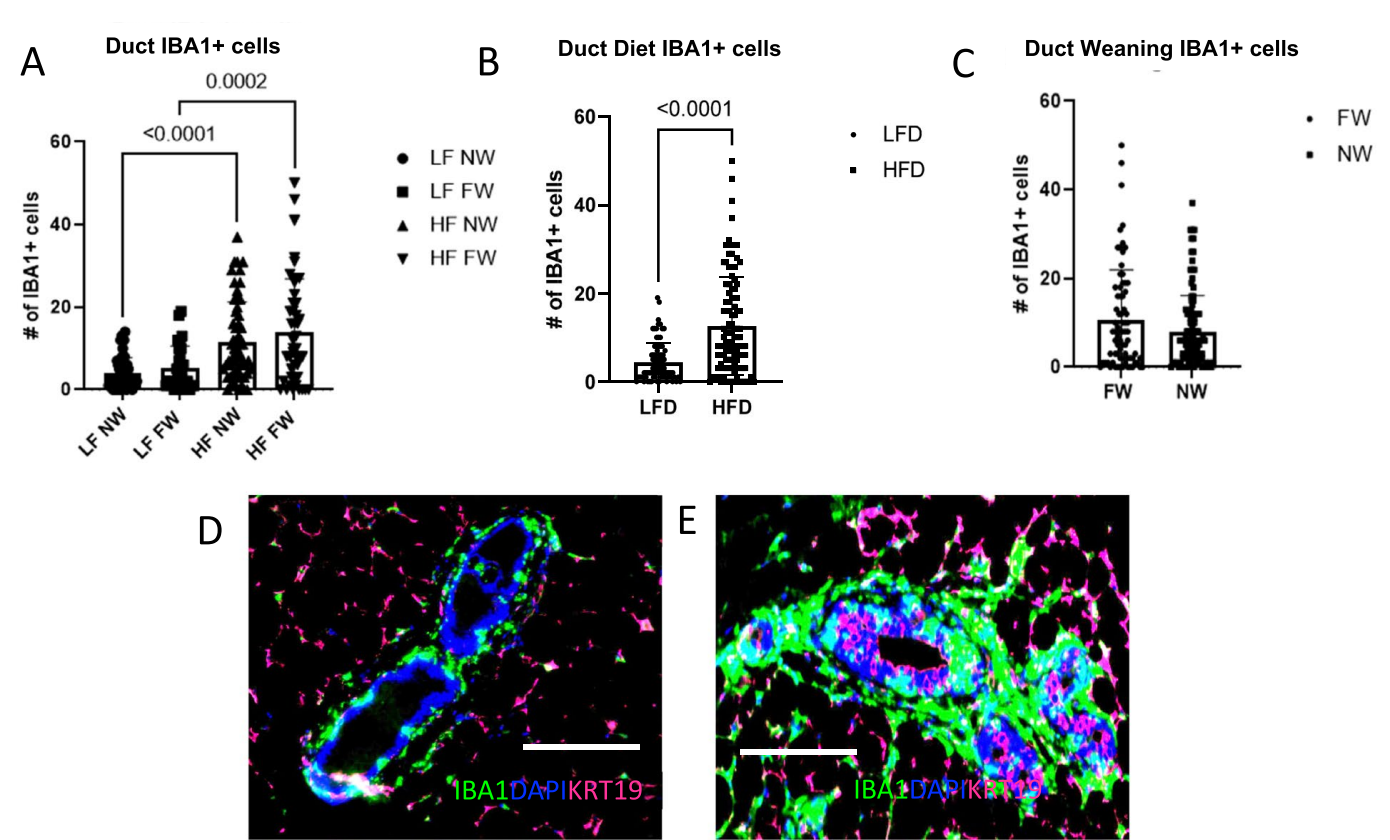

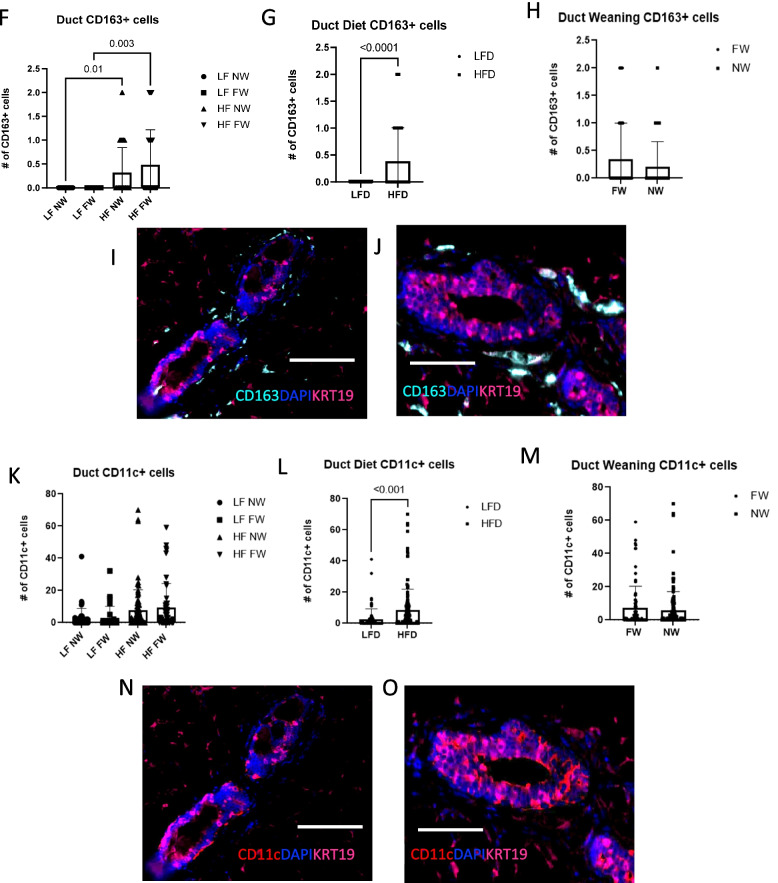
High-fat diet enhances inflammatory immune cells in the mammary ducts. **A**, **B**, **C** IBA1 expressing cells per duct. (D, E) Immunofluorescence (IF) images show IBA1^+^ cells (green) in postpartum mammary ducts from LFD mice (**D**) and HFD mice (**E**). **F**, **G**, **H**. CD163 expressing cells per duct. **I**, **J** IF images show CD163^+^ cells (teal) in postpartum mammary ducts from LFD mice (**I**) and HFD mice (**J**). **K**, **L**, **M** HFD led to increased numbers of ductal cells expressing the immune-cell activation marker CD11c independent of weaning status. **N**, **O** IF images show expression of ductal CD11c^+^ cells (red) in the ducts from HFD mice (**N**) and the LFD mice (**O**). All scale bars are 100 μm

HFD also increased the overall number of ductal IBA1+ macrophages (Fig. 7A; *P* < 0.0001 LF NW vs HF NW; *P* = 0.0002 LF FW vs HF FW) independent of weaning status (Fig. 7B, *P* < 0.0001; Fig. 7C, *P* = NS). These differences are apparent in the IF images of the postpartum mammary ducts from LFD vs HFD mice, in which IBA1+ cells in and around the ductal epithelium were more prevalent in the mice fed HFD (Fig. 7D, E). In addition, HFD mice had significantly higher numbers of CD163+ cells, independent of weaning status (Fig. 7F, *P* = 0.01, *P* = 0.003; Fig. 7G, *P* < 0.0001; and Fig. 7H, *P* = NS). These differences were evident in the IF images of postpartum mammary ducts from LFD vs HFD mice (Fig. 7I, J). Finally, the HFD increased the numbers of ductal CD11c+ cells (Fig. 7L, *P* < 0.001) but weaning status did not (Fig. 7M, *P* = NS). Again, ductal CD11c+ cells were more evident in the ducts from the HFD mice than in the ducts from the LFD mice (Fig. 7N, O).

## Discussion

Relatively few studies have been reported on lymphatic function in the mammary gland. Agollah and colleagues used near-infrared lymphatic imaging of immunocompromised mice in vivo for up to 11 weeks before inoculation of orthotopic SUM149 IBC tumor cells and again after inoculation. In that study, the lymphatic drainage patterns from the tumor-involved mammary gland in mice with IBC (SUM149) tumors changed such that lymphatic drainage was rerouted as a result of lymphatic obstruction during tumor growth [13]. Here we extend this work using this functional imaging technique to examine the contralateral mammary gland lymphatic function before and after tumor initiation to examine the systemic effects of the tumor independent of the tumor obstruction and to examine the infiltrates that may contribute to these systemic lymphatic effects of risk factors and tumors.

HFD increased lymphatic pulsing independent of parity and weaning and modestly increased growth of SUM149 IBC tumors. HFD increased markers of inflammation in the contralateral gland independent of lactation/weaning status. Thus, HFD, lymphatic pulsing, and tumor-induced inflammation are collectively correlated with increased tumor growth in this model. Although the timing of weaning (i.e., duration of nursing) did not affect lymphatic pulsing or tumor growth in this model, this is the first study to model the combination of these risk factors simultaneously and at a time point delayed until after pregnancy, as experienced by most patients. This is the first report that a HFD promoted xenograft growth of an IBC cell line (SUM149) and, contrary to our hypothesis, we found no effect of weaning timing on tumor growth in this model. All but one of the multiparous mice developed IBC-like skin -symptoms, which is increased relative to historical models of nulliparous mice with SUM149 tumors [9]. This may be attributed to pregnancy, but could also be age or other unmeasured factors. Lymphatic function pre-tumor was significanlty correlated with tumor size at eight weeks and was associated with increased infiltration by cells expressing the lymphatic trafficking ligand CCL21. HFD and FW were further associated with increased numbers of lymphangiogenic PDPN^+^ macrophages in the mammary ducts, highlighting one potential synergy in risk factors for further study. Finally, HFD also increased the numbers of IBA1^+^ and CD163^+^ macrophages and CD11c^+^ cells in mammary gland ducts. We suggest that further studies of the intersection of risk factors and mammary gland inflammatory infiltrates in increasing lymphatic function and promoting tumor growth are warranted.

To address the question of whether pre-tumor lymphatic function is a relevant endpoint with regard to tumor progression, we found that HFD increased lymphatic pulsing activity before tumor implantation to a degree similar to the significant increase in pulsing after inoculation of triple-negative IBC SUM149 tumor cells in LFD mice. We further found a significant correlation between pulsing and maximum tumor volume, suggesting that lymphatic pulsing before tumor development may contribute to tumor growth. This supposition has not been directly tested but contributes to the hypothesis that pre-tumor breast changes may influence the growth patterns and symptoms of some tumors that present as IBC.

We previously demonstrated in preclinical models that macrophage-educated mesenchymal stem cells promote IBC growth, and that inhibiting macrophage recruitment in vivo inhibited IBC tumor growth, tumor recurrence, and skin invasion [8]. Further, we found that normal breast tissue adjacent to IBC tumors was enriched with macrophage infiltration that was evident in the contralateral breast when those samples were available for review [14]. Macrophages are a dominant immune cell population in the mammary duct. A unique population of tissue-resident ductal macrophages form a tight network with the epithelium, which allows constant monitoring of the epithelium [15, 16]. There were no differences in the ductal macrophages based on weaning status in the multiparous mice, whereas HFD increased the numbers of IBA1^+^, CD163^+^, and CD11c^+^ cells within the mammary gland. A specialized subset of macrophages, PoEMs, is of special interest to lymphatic studies, as PoEMs can integrate into the lymphatic vasculature to promote neo-lymphangiogenesis as well as LVSI [17, 18]. In one study of patients with breast cancer, the association of PDPN-expressing macrophages with tumor lymphatic vessels correlated with increased lymph node and distant organ metastasis [17]. Another group found that among mammary tumor-infiltrating immune cells, the cells that expressed the highest levels of PDPN were tumor-associated macrophages. PDPN-expressing macrophages that are proximal to lymphatics stimulate local matrix remodeling and promote lymphatic vessel growth and lymphoinvasion [18]. The role of this macrophage subset in priming the stroma for lymphatic-invading tumor spread warrants further investigation.

Evidence for persistent post-pregnancy molecular changes in IBC comes from studies of involution gene signatures in normal adjacent breast tissues from patients with IBC and non-IBC [14]. Consistent with the a priori hypothesis, a signature specific to a stage of involution was enriched in the normal adjacent breast tissues of IBC patients who had undergone involution years earlier. These findings, in combination with epidemiologic studies implicating obesity and lack of breast feeding as risk factors in IBC [10], led us to hypothesize that forced weaning may enhance the persistence of a postpartum tumor-promoting microenvironment, one that could be promoted further by diet. However, we did not find the expected increase in tumor growth or lymphatic function based on weaning status, although several inflammatory subsets including PoEMs were associated with weaning status. We examined the expression of the chemokine receptor CCR7, which is expressed on mature leukocytes and T cells and induces leukocyte homing towards CCL21-expressing lymph nodes across a chemotaxis gradient [11, 19–22]. Identification of CCR7 expression in SUM149 cells prompts the hypothesis that tumor cells use this well-defined immune mechanism for lymphatic homing for LVSI. Melanoma studies have demonstrated that metastatic melanoma tumor cells express CCR7, which mediates chemotactic metastasis towards proximal lymphatics, resulting in lymphoinvasion [23, 24]. Here we found significant CCR7 expression across tumors of all groups and a correlation between infiltration of CCL21-expressing cells and lymphatic pulsing. Thus, CCR7 may be worthy of further mechanistic investigation. Considering the intersection with HFD, obesity can promote the accumulation of CCR7^+^ macrophages and dendritic cells in adipose tissue in close proximity to lymph nodes [25]; however, in this model we were not able to co-localize IBA1 and CCR7 (in two different staining panels) to assess this.

A limitation of the current study was the absence of nulliparous controls for tumor promotion and multiplex imaging. In addition, we did not look at the impact on metastasis. Others have reported that HFD or obesity causes lymphatic dysfunction characterized by the reduced ability to transport lymph, leaky vessels, and changes in the expression of lymphatic endothelial-cell markers [26, 27], as opposed to the increase in function we report here. However, one of those studies involved quantifying lymphatic contractile activity in the collecting vessels in the limb, near the entrance to popliteal lymph node [26]. The other focused on the effect of HFD-induced obesity in obesity-resistant and obesity-prone mouse strains. That study revealed that only obesity-prone mice—not obesity-resistant mice—that consumed HFD had impaired lymphatic function, increased perilymphatic inflammation, and altered lymphatic endothelial-cell gene expression [27]. The divergence between these findings and our own regarding the effects of obesity on lymphatic activity in mouse models could have several explanations, including the amount of time the mice were given the diet before imaging and the mouse strain used; ours are the first results in immunocompromised mice. Another contributing factor is which vessels were being assessed, because regional heterogeneity of lymphatic vessels has been reported to affect lymphatic contractile function [28]. Indeed, studies involving collecting lymphatic vessels from rats have shown regional variations in lymphatic contractile responses to physical stimuli under exposure to particular conditions and environments [29, 30]. We were not able to determine why imaging was not successful in some vessels in the diet alone experiment at the last timepoint so cannot conclude if this is a meaningful development, however this issue was not observed in the weaning and diet experiment.

In conclusion, we demonstrated for the first time that consumption of HFD increased lymphatic function independent of weaning status in this immunocompromised mouse model. We also report for the first time that HFD promoted the growth of SUM149 IBC tumor cells in these mice and pre-tumor lymphatic pulsing correlated to tumor size. We showed that tumor initiation prompted increases in lymphatic pulsing activity in LFD mice to an extent similar to that induced by HFD before tumor initiation. The increase in lymphatic pulsing activity and functionality was independent of lymphatic vessel density. We further demonstrated that HFD promoted an inflammatory microenvironment, indicated by the presence of immune cells such as macrophages, M2 macrophages, and dendritic cells within the mammary ducts. Numbers of PDPN^+^ macrophages were also elevated in ducts of the mammary gland, to the greatest extent in the HFD FW group. These persistent changes in the microenvironment and their influence on inflammatory infiltrates including CCL21 with regard to lymphatic function and promotion of tumor growth warrant further study.

### Supplementary Information

**Additional file 1:** **Supplementary Figure 1.** Schematic for testing the effects of diet on lymphatic function in nulliparous mice by using *in vivo* near-infrared fluorescence lymphatic imaging. **Supplementary Figure 2.** Schematic for testing the effects of diet and time of weaning on lymphatic function in multiparous mice by using *in vivo* near-infrared fluorescence lymphatic imaging. **Supplementary Figure 3.** Findings from multiplex immunofluorescence–stained IBC (SUM149) tumor sections from multiparous mice.
