# Supplementary material for: High-fat diet, but not duration of lactation, increases mammary gland lymphatic vessel function and subsequent growth of inflammatory breast cancer cells
Source: J Mammary Gland Biol Neoplasia. 2023 Oct 6;28(1):21. doi: 10.1007/s10911-023-09548-8 (PMC10558390; doi:10.1007/s10911-023-09548-8)
Supplement: Supplementary file 1 — Additional file 1: Supplementary Figure 1. Schematic for testing the effects of diet on lymphatic function in nulliparous mice by using in vivo near-infrared fluorescence lymphatic imaging. Supplementary Figure 2. Schematic for testing the effects of diet and time of weaning on lymphatic function in multiparous mice by using in vivo near-infrared fluorescence lymphatic imaging. Supplementary Figure 3. Findings from multiplex immunofluorescence–stained IBC (SUM149) tumor sections from multiparous mice. [file 10911_2023_9548_MOESM1_ESM.docx]

**SUPPLEMENTARY MATERIAL**


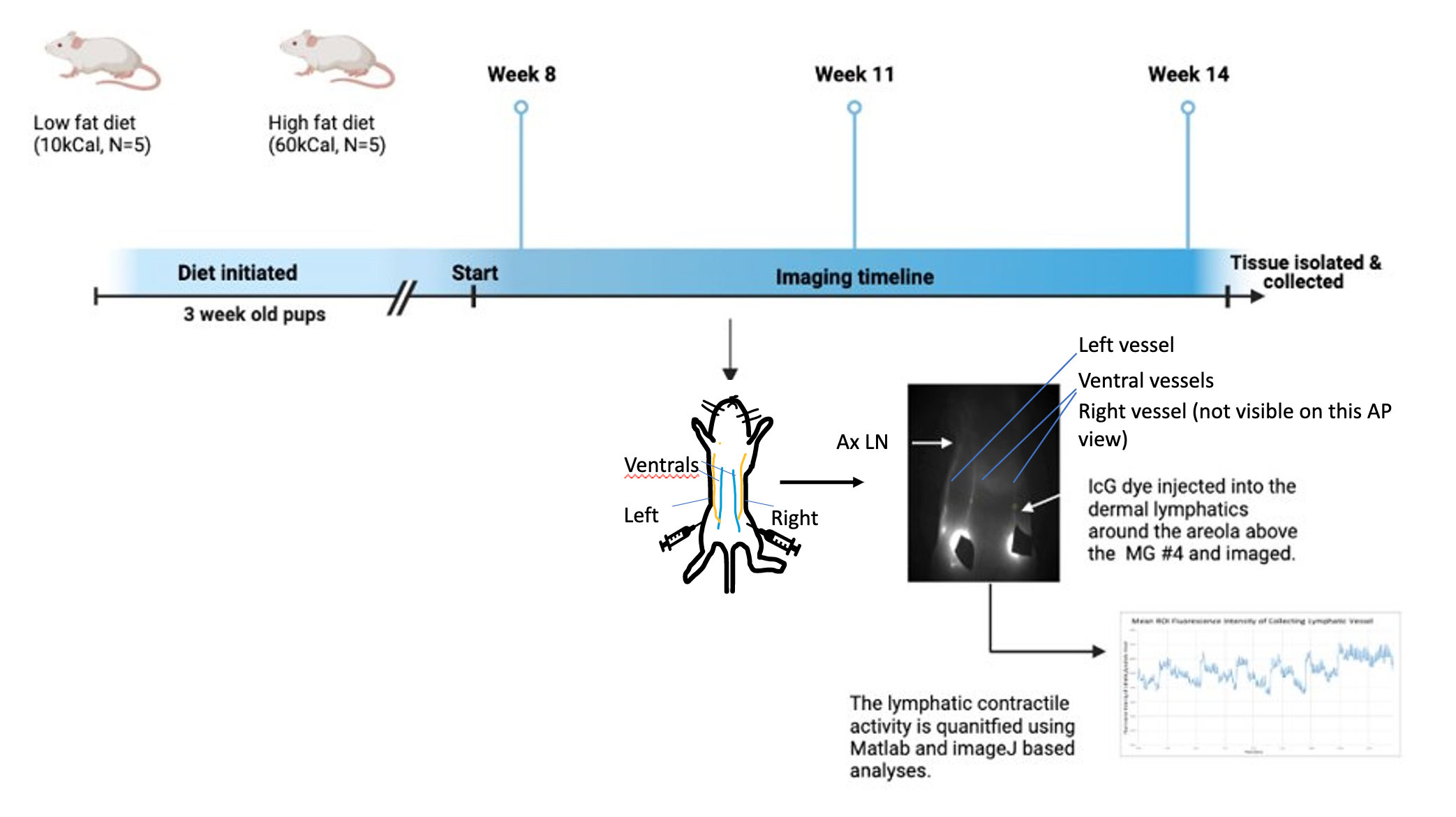


**Supplementary Figure 1. Schematic for testing the effects of diet on lymphatic function in nulliparous mice by using *in vivo* near-infrared fluorescence lymphatic imaging.** We measured lymphatic pulsing in nulliparous mice started on diets at 3 weeks of age. Indocyanin green dye drainage injected into the bilateral mammary glands was assessed from mean fluorescence intensity graphs by *in vivo* near-infrared fluorescence imaging in lymphatics (ventrals, right, and left) at three timepoints (8, 11, and 14 weeks after initiation of diet). Two left and right ROIs were assessed for each mouse, both ventral lymphatics were assessed.


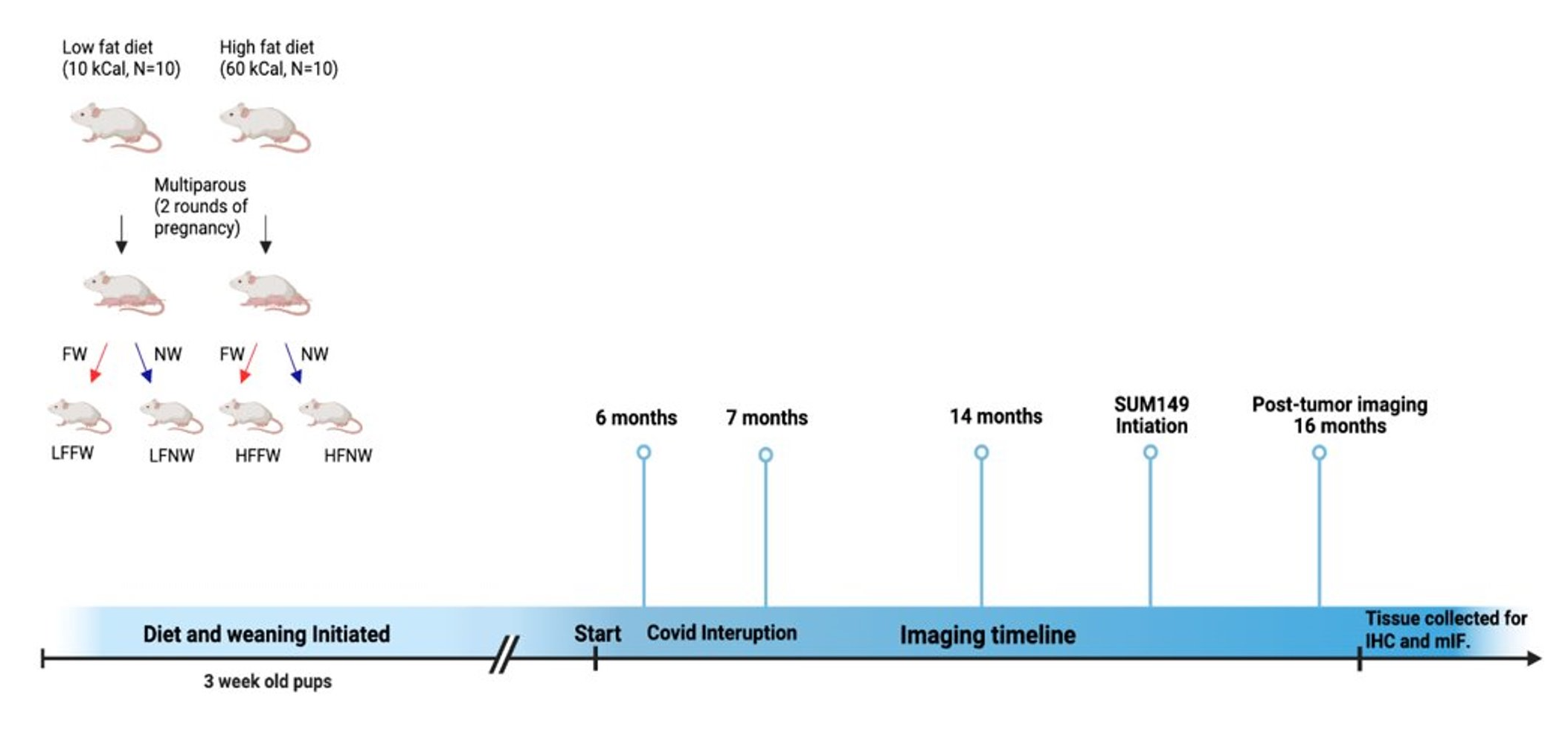


**Supplementary Figure 2. Schematic for testing the effects of diet and time of weaning on lymphatic function in multiparous mice by using *in vivo* near-infrared fluorescence lymphatic imaging.** The potential synergistic effects of a diet (high-fat, HFD; low-fat, LFD) and the timing of weaning (i.e., duration of lactation) on dermal lymphatic pulsing activity were investigated in multiparous mice that had been fed either HFD or LFD and either abruptly (force) weaned (FW) or naturally (nurse) weaned (NW). Baseline images were obtained at 6-7 months and 14 months (timeline interrupted by Covid-19 pandemic), ventral vessels only. Tumors were then inoculated followed by re-imaging at 16 months.


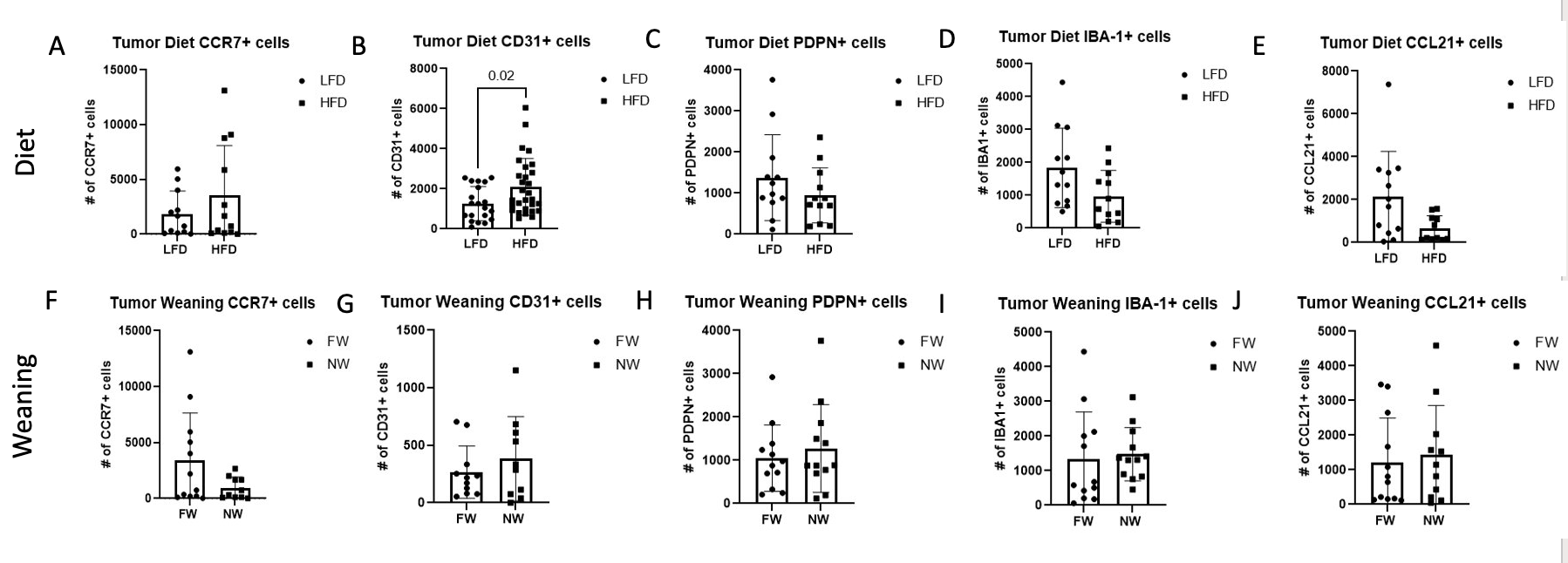


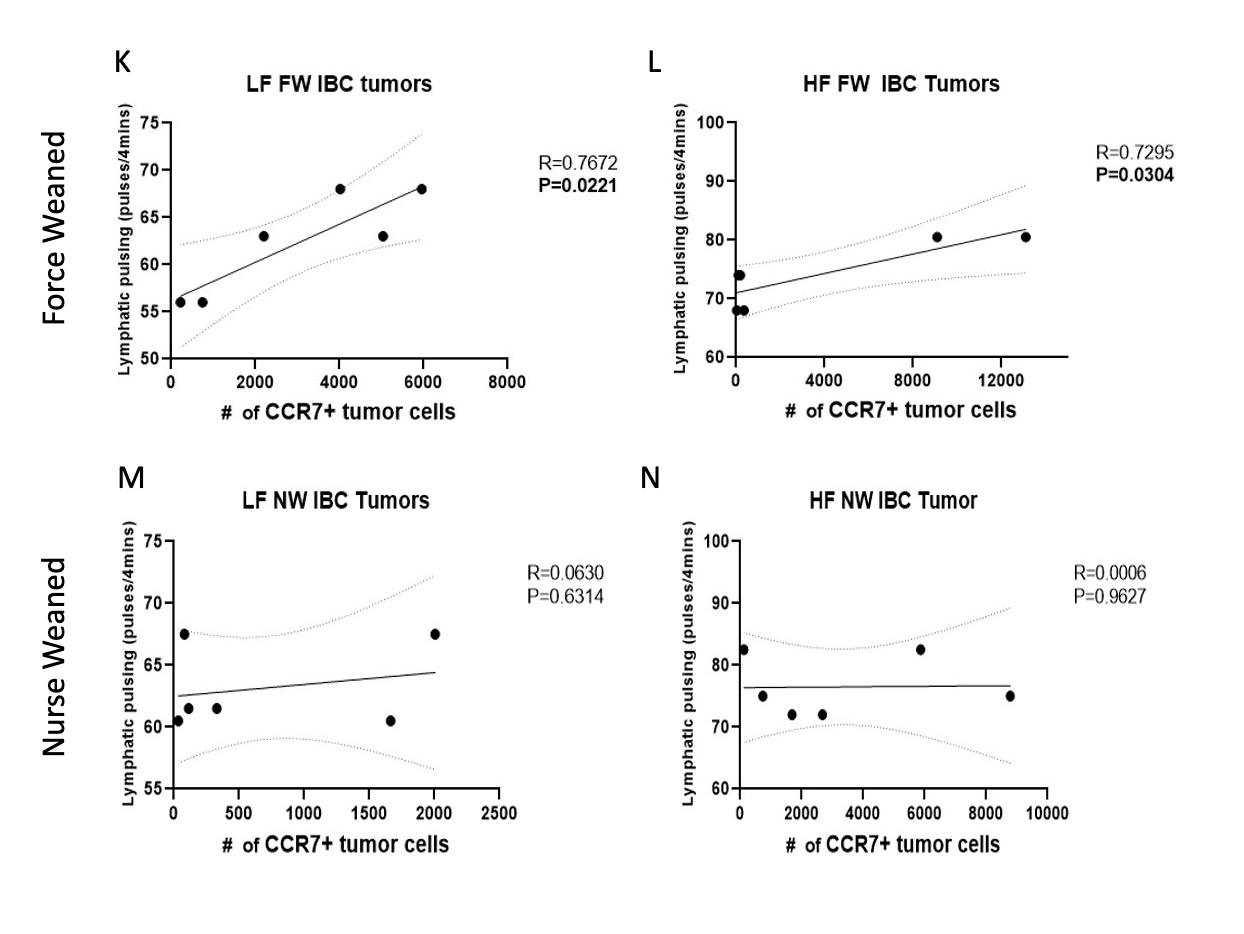


**Supplementary Figure 3. Findings from multiplex immunofluorescence–stained IBC (SUM149) tumor sections from multiparous mice.** Percent of tumor cells (KRT19 positive staining in tumor) was compared between LF versus HF mice for CCR7, CD31, PDPN, IBA1, and CCL21 expression (A-E, respectively) and between NW versus FW mice for the same markers (F-J, respectively). Where p values are not indicated they were < 0.05. (K,L) Correlation analysis of CCR7^+^ in IBC (SUM149) tumor cells and pre-tumor lymphatic pulsing rates were performed and examined by nursing and diet group (LF and HF FW, K, L; LF and HF NW M,N).
